# Narrative Review on Parathyroid Gland Disorders in Individuals Living with HIV: An Update

**DOI:** 10.3390/metabo15110704

**Published:** 2025-10-29

**Authors:** Ahmed Hassan, Yashar Mashayekhi, Ridwan Hashi, Musaab Ahmed, Dushyant Mital, Mohamed H. Ahmed

**Affiliations:** 1 Faculty of Medicine, Alexandria University, Alexandria 5424041, Egypt; ahmed.mohamed2133@alexmed.edu.eg; 2 Department of Orthopaedic, Leicester University Hospital, Leicester LE1 5WW, UK; yashar.mashayekhi1@nhs.net; 3 Department of Surgery, Norforlk and Norwich University Hospital, Norwich NR4 7UY, UK; Ridwan.Hashi@nnuh.nhs.uk; 4 College of Medicine, Ajman University, Ajman P.O. Box 346, United Arab Emirates; m.omer@ajman.ac.ae; 5 Department of Blood Borne Virus and HIV, Milton Keynes University Hospital NHS Foundation Trust, Eaglestone, Milton Keynes MK6 5LD, UK; dushyant.mital@mkuh.nhs.uk; 6 Department of Geriatric Medicine, Milton Keynes University Hospital NHS Foundation Trust, Eaglestone, Milton Keynes MK6 5LD, UK; 7 Department of Medicine and HIV Metabolic Clinic, Milton Keynes University Hospital NHS Foundation Trust, Eaglestone, Milton Keynes MK6 5LD, UK; 8 Faculty of Medicine and Health Sciences, University of Buckingham, Buckingham MK18 1EG, UK

**Keywords:** HIV, parathyroid hormone (PTH), combined antiretroviral therapy (cART), tenofovir disoproxil fumarate (TDF), vitamin D, hypoparathyroidism, hyperparathyroidism, secondary hyperparathyroidism

## Abstract

Parathyroid gland disorders, including secondary hyperparathyroidism, have emerged as significant endocrine complications in people living with HIV (PLWHIV). This narrative review synthesises recent evidence on the prevalence, mechanisms, and clinical implications of parathyroid dysfunction in PLWHIV. HIV infection, combined antiretroviral therapy (cART), and immune activation contribute to parathyroid dysfunction, with cART regimens, particularly Tenofovir Disoproxil Fumarate (TDF), exacerbating these disturbances by altering the calcium and parathyroid hormone (PTH) dynamics. Studies show that PTH levels in PLWHIV on TDF were significantly elevated compared to those on non-TDF-based cART regimens. Histopathological studies highlight a higher prevalence of parathyroid hyperplasia in PLWHIV, often linked to chronic deficiencies in calcium, magnesium, and vitamin D, as well as immune dysregulation. The dysfunction observed ranges from inappropriate elevation of PTH levels to hypoparathyroidism, leading to rapid bone density loss and an increased fracture risk. Despite the fact that HIV is a condition associated with high malignancy, parathyroid malignancy is a very rare issue. Despite the growing recognition of these complications, routine screening for PTH and bone health remains inadequate in standard clinical HIV care. This review advocates for incorporating routine monitoring of serum PTH, calcium, phosphate, and vitamin D levels, especially in those on TDF-based cART. Early detection of subclinical parathyroid dysfunction can prevent complications such as secondary hyperparathyroidism and neuromuscular symptoms. Clinicians should be aware of atypical biochemical presentations, such as elevated PTH with normal calcium, which may indicate cART-induced dysregulation, improving patient management and outcomes.

## 1. Introduction

HIV appears to interact with all endocrine glands, including the parathyroid gland. It is well established that parathyroid hormone (PTH) plays a central role in maintaining calcium and phosphate homeostasis through its actions on bone, kidney, and the gastrointestinal tract via activation of vitamin D. Its secretion is regulated by the calcium-sensing receptor (CaSR) located on parathyroid cells [1,2,3]. Therefore, low or insufficient secretion or action of PTH leads to hypoparathyroidism; biochemically, this will show as low PTH, low calcium, and high phosphate [1]. Primary hyperparathyroidism, more common and appears to be characterised by autonomous overproduction (adenoma or hyperplasia of the parathyroid gland) of PTH, leading to high plasma calcium and low plasma phosphate, and increased bone resorption with possible increase in urinary calcium excretion [2]. Importantly, familial hypocalciuric hypercalcaemia (FHH) is caused by inactivating mutations of the CaSR or related signalling pathways, which raise the calcium set-point required to suppress PTH secretion. The condition presents with lifelong mild to moderate hypercalcaemia, inappropriately normal or slightly elevated PTH, and hypocalciuria [3]. The CaSR serves as the molecular “gatekeeper” for calcium homeostasis—high extracellular calcium activates the receptor, leading to suppression of PTH secretion. When the CaSR function is disrupted, as in FHH, this negative feedback is blunted, resulting in persistently elevated calcium levels [3]. Therefore, in PLWHIV, urinary calcium excretion will aid in the need to exclude FHH and to proceed to genetic testing.

It is recognised that parathyroid gland disorders, along with hyperparathyroidism, as major endocrine complications affecting people with PLWHIV. The development of these conditions results from three main factors, which include cART-induced metabolic shifts, persistent immune activation, and widespread vitamin D insufficiency. In PLWHIV, the clinical burden has largely transitioned from opportunistic infectious diseases to age-related chronic non-communicable diseases, including metabolic and bone disorders, partially due to potent cART regimens like cabotegravir, which largely extend life expectancy [4,5,6,7].

Ahmed et al. (2023) reported that older PLWHIV in a UK metabolic HIV clinic frequently present with metabolic syndrome and early endocrine dysfunction, including hyperparathyroidism, underscoring parathyroid disruption as part of systemic metabolic dysregulation [4,5]. Madanhire et al., in a cohort of 842 HIV-positive adolescents from Southern Africa, demonstrated that PTH levels only stabilise once serum vitamin D exceeds approximately 75 nmol/L, higher than conventional thresholds indicating recalibrated calcium–PTH dynamics in PLWHIV [8]. In a case report, it was shown that primary hypoparathyroidism occurs rarely after COVID-19 infection, and this was attributed to COVID-19-related immune or inflammatory mechanisms, direct viral damage to parathyroid glands, or disruption of calcium homeostasis may underlie this dysfunction [9]. In this narrative review, we will provide evidence that PLWHIV can develop both hyper and hypoparathyroidism [10]. PLWHIV who do not receive treatment for parathyroid dysfunction experience rapid bone density loss and muscle weakness and develop fragility fractures at a rate six to seven times higher than HIV-negative individuals, but at an age ten years earlier than expected [8,11].

**Figure 1:**
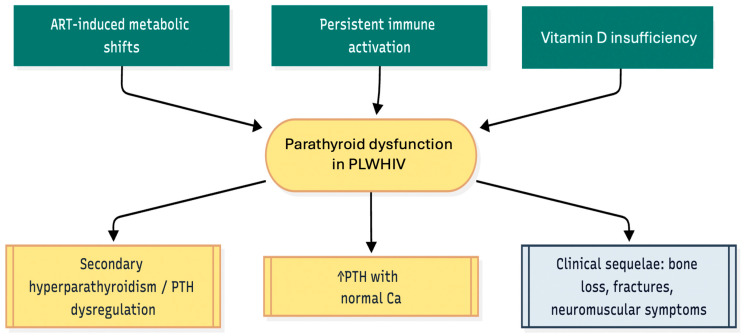
cART-induced metabolic shifts, persistent immune activation, and vitamin D insufficiency converge to produce parathyroid dysfunction in PLWHIV, manifesting as secondary hyperparathyroidism, atypical biochemistry (e.g., elevated PTH with normal calcium), and downstream skeletal/neuromuscular sequelae. (PTH, parathyroid hormone; cART, combined antiretroviral therapy, the arrow inside the figure indicates increased PTH).

The majority of HIV care guidelines lack routine PTH or bone-health screening despite strong evidence, which creates a significant care gap. This review combines recent epidemiological findings with new mechanistic knowledge and treatment progress that includes specific vitamin D/calcium supplements, cART adjustment methods, and innovative parathyroid-directed therapies. This review aims to establish an evidence-based framework for early detection and treatment of parathyroid disorders in PLWHIV while enhancing endocrine resilience in contemporary HIV care [6,7]. The review summarises epidemiology, pathophysiology (immune dysregulation and effects of antiretroviral therapy), and clinical consequences of parathyroid dysfunction among PLWHIV, specifically PTH dysregulation. It is limited to studies published in English, in full text from 1982 to 2025.(Figure 1). 

## 2. Methods and Materials

The methodology adopted for this narrative review included a literature search conducted in four electronic databases: PubMed, Medline, Scopus, and Google Scholar. These four databases were chosen for the review because of their widespread coverage of biochemical and clinical literature. The primary aim of the search strategy on these platforms was to identify the relationship between parathyroid disorders and HIV. The keywords used to identify a range of relevant literature included (“Parathyroid gland” OR “Hypoparathyroidism” OR “Hyperparathyroidism”) AND (“HIV” OR “Acquired Immunodeficiency Syndrome” (AIDS). These keywords aimed to synthesise any studies exploring the relationship between the parathyroid gland and HIV, including case reports and histological studies.

**Figure 2:**
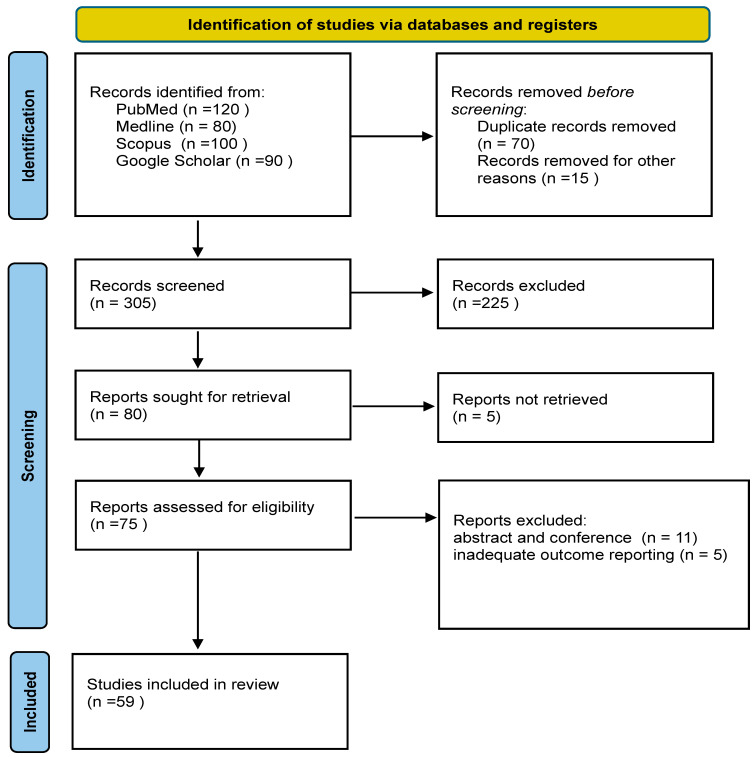
PRISMA diagram showing steps taken to conduct this narrative review.

The inclusion criteria for this review were studies published in the English language and with full text available. Inclusion criteria: peer-reviewed studies in English with full text available that examined parathyroid function in PLWHIV, including clinical, biochemical, or pathological outcomes (e.g., PTH, calcium, phosphate, vitamin D, or histopathology/autopsy of parathyroid tissue), case reports/series, and observational or interventional studies on cART effects on the calcium–PTH axis. Exclusion criteria: non-HIV populations; non-English publications; grey literature or unavailable full texts; studies focused solely on skeletal outcomes without parathyroid measures; conference abstracts only; and overlapping datasets. As this was a narrative review and due to the limited number of publications in the field, it was difficult to develop a formal protocol or apply a structured assessment of study quality or risk of bias, which would have increased transparency and rigour. Consequently, the review incorporates a wide range of evidence, including case reports, observational studies, and clinical trials. The final selection comprised 59 studies; Approximately 34% are reviews, narrative papers, or systematic reviews and meta-analyses. Around 31% are cohort, cross-sectional, or other observational studies, examining clinical associations between HIV, vitamin D, and parathyroid hormone. Randomised controlled trials account for about 12%, mainly focusing on vitamin D and calcium supplementation or antiretroviral therapy-related bone effects. Case reports and case series make up roughly 14%, illustrating rare presentations such as hypoparathyroidism or hypocalcemia in HIV infection. In addition, about 7% are basic or mechanistic studies exploring molecular pathways, while 2% represent doctoral or non-journal research (Figure 2).

## 3. HIV and the Parathyroid Gland

The parathyroid gland consists of four small structures located posterior to the thyroid gland. Its primary function is the regulation of calcium homeostasis through the secretion of PTH. In response to low circulating calcium levels, the gland releases PTH, which acts on osteoclasts to mobilise calcium from bone and increases renal calcium reabsorption to restore serum calcium levels [10]. In HIV infection, parathyroid gland function may be compromised by various factors, including infections, immune response, the impact of cART, low vitamin D, and magnesium [11]. Investigations into the involvement of parathyroid cells have reported that the cells can express receptors with a similar structure to those of CD4 molecules. This may be the explanation of the early symptomatic hypoparathyroidism in PLWHIV at early levels of the infection, when there is still high viral load [12]. The study of Hellman et al. (2000), with the aid of the monoclonal antibody anti-Leu3a, which binds the CD4 molecules, could support the role of this theory because their study showed that the parathyroid cells exhibited a positive staining of the CD4 molecules on the surface [13]. Such findings showed that the immune system could have also attacked parathyroid cells through anti-CD4 (antibody) binding antibodies, and most likely, the parathyroid gland itself was directly infected [13]. The incidence of parathyroid dysfunction in PLWHIV differs across continents, and some regions have had higher rates attributed to cART regimens and substantial chronic immune activation. Importantly, Table 1 discusses the most significant processes that lead to parathyroid abnormalities in PLWHIV, which are immune dysregulation, the lack of vitamin D, and the effect of a special cART regimen component, TDF. Importantly, while there is limited data on the prevalence of primary hyperparathyroidism due to parathyroid adenoma in individuals with HIV, current evidence does not indicate a higher incidence of this condition in the HIV-positive population compared to the general population.

For further indications of disturbed parathyroid in HIV, the data provided by Jaeger et al., who measured PTH secretion in six patients with AIDS (CD4 < 50/L) and ten control individuals during hypocalcaemia induced by EDTA infusion, gave further evidence of altered parathyroid behaviour in HIV [14]. The study demonstrated that both baseline serum PTH levels were lowered in PWLH (*n* = 6; 14.2 ± 2.1 ng/L) compared to control groups (*n* = 10; 23 ± 10 ng/L). Post EDTA-induced hypocalcaemia, maximal PTH response remained significantly lower in the HIV-positive group compared to the control group (*p* < 0.04), suggesting impaired parathyroid function in AIDS [14]. Hellman et al. also observed similar results of decreased PTH levels in patients with HIV infection (*n* = 38; 13.9 ± 2.3 ng/L) compared to normal individuals (*n* = 38; 38.1 ± 3.1 ng/L) [13].

**Figure 3:**
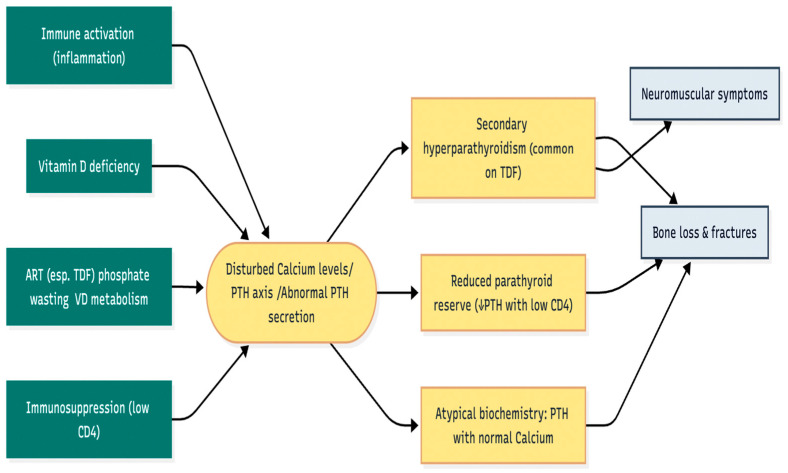
Immune activation, vitamin D deficiency, TDF-related effects (phosphate wasting/altered vitamin D metabolism), and immunosuppression (low CD4) collectively dysregulate the calcium–PTH axis, yielding phenotypes such as secondary hyperparathyroidism, reduced parathyroid reserve, and atypical laboratory profiles that predispose to bone loss and neuromuscular symptoms. (TDF, tenofovir disoproxil fumarate).

Although HIV-related hypoparathyroidism is rare in the current literature, it has been documented in several case reports. These cases explore whether HIV affects the parathyroid gland directly or indirectly by impairing calcium-regulatory pathways and altering PTH levels [15]. While vitamin D deficiency is often linked to hypocalcaemia in PLWHIV, these case reports challenge this association. The initial case report is by Gulden et al., reporting the case of a 67-year-old male living with HIV who presented with severe symptomatic hypocalcaemia (ionised calcium of 0.98 mmol/L) and primary hypoparathyroidism (PTH of 1.30 pmol/L) despite having sufficient vitamin D levels [15]. The authors ruled out other causes such as neck surgery and autoimmune disease, suggesting that the parathyroid insufficiency was likely secondary to HIV infection, which resulted in the hypocalcaemia observed in this patient [15]. This supports the conclusions of Sandhu et al. that described the case of a 54-year-old HIV-positive female patient with a two-year history of persistent hypocalcaemia with a total calcium of 5.7 mg/dL (range: 8.4–10.2 mg/dL) and PTH levels of 7.6 pg/mL (range: 15–65 pg/mL) [16]. The coexistence of hypocalcaemia, hyperphosphatemia, and suppressed PTH levels is indicative of primary hypoparathyroidism, which, in the absence of identifiable secondary causes, suggests a potential direct role of HIV in the glandular dysfunction [16]. Several case reports have documented parathyroid dysfunction in HIV-positive individuals, shedding light on its clinical manifestations and potential causes. Table 2 summarises the findings from these case reports, offering insights into the unique presentations and diagnostic challenges of parathyroid disorders in PLWHIV. It is possible to suggest that, until further research clarifies the actual impact of HIV on the parathyroid gland, it remains crucial to assess parathyroid function and vitamin D status in people living with HIV (PLWH) who present with myopathy and hypocalcaemia. It is not yet clear whether viral suppression and an adequate CD4 count provide protection to the parathyroid gland. This may also lead to whether the parathyroid dysfunction associated with high viral load may explain in part the high risk of osteoporosis and fracture that can occur with high viral load or at the time of the diagnosis of HIV. Therefore, further research at the cellular, molecular, and population levels, as well as clinical studies, is required. (Figure 3)

## 4. Histological and Autopsy Evidence of HIV-Related Parathyroid Dysfunction

Impaired parathyroid function in people living with HIV (PLWHIV) has been documented in several clinical studies; however, the underlying mechanisms remain incompletely understood, warranting further histological and autopsy-based investigations to elucidate the pathophysiology. In a retrospective autopsy study, Cherqaoui et al. examined 102 HIV-positive African American patients and found a markedly higher prevalence of parathyroid hyperplasia compared with control subjects (22.6% vs. 2.6%, *p* < 0.04) [17]. While these findings indicate a potential association between HIV infection and parathyroid morphological changes, the authors postulated that the hyperplasia was more likely secondary to chronic hypocalcaemia and vitamin D deficiency, both of which are highly prevalent among African American individuals with HIV [17]. A more nuanced interpretation of these findings, however, raises the question of whether parathyroid hyperplasia represents a direct viral effect or a compensatory response to systemic metabolic disturbances. It is plausible that the chronic inflammation and immune activation seen in advanced HIV infection may indirectly modulate parathyroid function through cytokine-mediated pathways or renal impairment. Furthermore, nutritional deficiencies and renal dysfunction—both common in PLWHIV—may exacerbate secondary hyperparathyroidism, blurring the distinction between primary endocrine involvement and secondary metabolic adaptation. Similarly, Kühne et al.’s comprehensive review on the endocrine effects of HIV infection reported a progressive decline in parathyroid hormone (PTH) secretion in association with decreasing CD4 counts, suggesting a possible link between viral burden and glandular dysfunction. Importantly, their review also highlighted histopathological evidence of parathyroid infiltration and destruction in patients with advanced HIV and disseminated opportunistic infections, such as cytomegalovirus and Pneumocystis carinii, particularly affecting the neck region [11,18]. Together, these observations imply that parathyroid abnormalities in HIV may reflect a multifactorial process—arising from the interplay of direct viral injury, opportunistic infection, and systemic metabolic disturbances rather than a singular pathological mechanism.

## 5. Impact of cART on Parathyroid Gland Dysfunction

The introduction of cART, particularly TDF, has significantly enhanced the clinical management and life expectancy of individuals living with HIV. However, its use has raised concerns regarding endocrine disturbances, particularly in the regulation of PTH [19,20,21]. Cross-sectional data analysis of the effect of TDF-based cART regimens on endocrine functions observed secondary hyperparathyroidism in patients on TDF with low vitamin D [20,21]. Masiá et al. conducted a longitudinal study assessing changes in PTH and vitamin D levels among 51 individuals initiating antiretroviral therapy, comparing two treatment regimens: TDF/Emtricitabine (*n* = 31) and Abacavir/Lamivudine (*n* = 26) [19]. Patients on TDF showed significantly elevated PTH levels at weeks 4 (*p* = 0.01), 24 (*p* = 0.008), and 36 (*p* = 0.02), exceeding the upper normal limit from weeks 24 to 48. Despite this, no differences were observed in vitamin D, calcium, or phosphate between treatment groups. Multivariable analysis confirmed TDF/emtricitabine as an independent predictor of elevated PTH (≥53 ng/L) [19]. TDF has been shown to impact parathyroid function by elevating parathyroid hormone levels. Table 3 evaluates the effects of various cART regimens on PTH levels and the associated clinical implications. Further data from Klassen et al. and Noe et al. both demonstrate a consistent association between a TDF-based cART regimen and elevated PTH levels [22,23]. Klassen et al.’s cohort study of 56 HIV-1-infected patients reported an increase in PTH among patients on TDF independent of serum calcium and vitamin D status in non-white male patients compared to the similar group on non-TDF (mean difference 3.1 pmol/L, 95% CI 5.3 to 0.9; *p* = 0.007) [22]. Similarly, Noe et al. reported persistent PTH elevation in all quartiles of corrected calcium for PLWHIV on TDF compared to those on non-TDF regimens [24]. Taken together, these studies underscore a consistent pattern of PTH elevation in patients undergoing TDF-based cART regimens, occurring independently of vitamin D status or calcium levels. Given these findings, routine monitoring of PTH and calcium levels may be warranted in patients receiving long-term TDF-based ART [25]. Importantly, the prevalence of secondary hyperparathyroidism was found to be 16.9% in a total of 1263 PLWHIV. Multivariable logistic regression modelling showed significant associations with elevated PTH for African ethnicity, low 25-hydroxyvitamin D levels, low calcium levels, and use of TDF, significant association between the use of TDF and secondary hyperparathyroidism [26].

Noe et al. also showed parathyroid dysregulation is likely to be attributed to such a pattern in TDF-containing therapy [25]. The same authors also showed that higher levels of PTH seem to be needed to maintain normal calcium levels in PLWHIV on TDF-containing cART compared to non-TDF-containing cART. Optimal concentrations for 25-hydroxy vitamin D and calcium might, therefore, be different in people using TDF than expected from general populations, but also in people living with HIV with non-TDF-containing cART. This might require different supplementation strategies but warrants further investigation [20]. Interestingly, Van Welzen showed that serum PTH levels drop significantly after the switch from TDF to TAF. They suggested that TDF-related bone loss is PTH-driven and that this effect is dose-dependent [26]. Furthermore, TDF was shown to inhibit the activity of calcium-sensing receptors, CaSR, in a dose-dependent manner and may, in part, explain that TDF-mediated hyperparathyroidism may be promoted by the direct effect of the drug on CaSR [27].

Across the included studies in Table 4, consistent signals emerge: PTH dysregulation in PLWHIV arises from immune activation and antiretroviral effects with TDF often linked to higher PTH despite normal calcium and 25(OH)D; histopathology reports describe parathyroid hyperplasia, and physiological work indicates reduced parathyroid reserve with CD4 cell loss [Table 4].

## 6. Impact of HIV on Vitamin D Metabolism

Vitamin D deficiency is highly prevalent amongst PLWHIV compared with the general population. This may be attributed to several factors, including the influence of the virus itself and the effects of cART on vitamin D metabolism. Such biochemical changes have important implications for bone health, immune function, and disease progression. Other contributing factors include chronic inflammation, altered sun exposure, and nutritional deficiencies. For example, a recent Chinese cross-sectional study showed that even HIV exposure without infection was associated with significantly lower vitamin D levels [28]. This supports the notion that HIV exposure alone may affect vitamin D status.

The mechanism by which HIV influences vitamin D metabolism is complex and not fully understood. Vitamin D appears to modulate immune responses partly by regulating autophagy and T-cell metabolism. In vitro, hormonally active vitamin D3 [1α,25(OH)_2_D_3_] was shown to induce autophagy in macrophages, resulting in reduced HIV-1 replication [29]. More recently, vitamin D was demonstrated to reduce CD4^+^ T-cell susceptibility to HIV infection by downregulating AKT phosphorylation and glucose uptake [30]. These findings suggest that vitamin D deficiency may play an important role in viral replication and negatively affect immune competence.

Several studies have highlighted the impact of combination cART on vitamin D metabolism. Efavirenz (EFV) induces cytochrome P450 enzymes, which accelerate vitamin D catabolism. In vitro fibroblast studies demonstrated that EFV upregulates CYP24A1 (responsible for catabolism) and downregulates CYP2R1 (responsible for activation), leading to lower circulating 25(OH)D levels [31]. Clinically, EFV use has consistently been associated with vitamin D deficiency. TDF can cause renal phosphate wasting and secondary hyperparathyroidism, further compounding vitamin D-related bone effects. Case series and clinical trials confirm that vitamin D deficiency exacerbates these risks. Therefore, monitoring vitamin D status is particularly important in PLWHIV receiving EFV or TDF-containing regimens.

Vitamin D deficiency in HIV has been linked to reduced bone mineral density (BMD), an increased risk of osteopenia and osteoporosis, and possibly worsen HIV disease progression. In the large EuroSIDA cohort, vitamin D deficiency was associated with faster clinical progression and higher mortality [32]. Bone loss following cART initiation is well documented. Randomised controlled trials have shown that vitamin D and calcium supplementation can mitigate this effect: in Thai adults on EFV/TDF-based regimens, supplementation reduced BMD loss compared with controls [33]; in adults initiating cART, supplementation attenuated both hip and spine bone loss [34]; and in adolescents and young adults with HIV receiving TDF, vitamin D_3_ significantly increased BMD [35]. These data strongly support routine vitamin D assessment and supplementation in high-risk groups.

Most interventional studies in PLWHIV have used 2000–4000 IU/day of vitamin D_3_ plus calcium, which effectively improved 25(OH)D levels, lowered parathyroid hormone (PTH), and attenuated cART-associated bone loss. International reviews now recommend aligning HIV care with general population vitamin D guidelines but maintaining a lower threshold for testing and supplementation in PLWHIV, particularly those treated with EFV or TDF [36]. This is due to the high prevalence of fractures in men and women living with HIV, especially elderly individuals with HIV and decreased nutritional intake [37,38,39].

The analytical considerations of vitamin D in HIV are crucial, as they also open up important research questions. One key issue is assay variability and accuracy. Most clinical studies have measured total 25-hydroxyvitamin D [25(OH)D]. However, immunoassays, which are widely used, may suffer from cross-reactivity and either under- or over-estimation compared with liquid chromatography–tandem mass spectrometry (LC-MS/MS), which is considered the current gold standard [40].

Standard testing quantifies the total 25(OH)D bound to vitamin D-binding protein (DBP) and albumin. Yet in PLWHIV, where chronic inflammation, cART effects, and altered protein levels are common, the free or bioavailable vitamin D fraction may better reflect biological activity [41]. Few studies in PLWHIV have measured free vitamin D, leaving uncertainty about whether deficiency thresholds based solely on total 25(OH)D are appropriate [33,34,41,42].

The biologically active form, 1,25-dihydroxyvitamin D [1,25(OH)_2_D], is rarely measured in practice because of its short half-life and tight physiological regulation. However, HIV-related immune activation, renal effects of TDF, and cART-related enzyme induction (e.g., CYP24A1) may alter the conversion between 25(OH)D and 1,25(OH)_2_D—changes that are often not captured in routine assays [42]. Additionally, inter-laboratory differences contribute to variability, making it difficult to compare vitamin D prevalence across HIV cohorts.

In PLWHIV, TDF-related renal tubular dysfunction, phosphate wasting, and secondary hyperparathyroidism can further complicate the interpretation of “low vitamin D” levels [43]. Without concurrent measurement of these markers, the true clinical relevance of vitamin D deficiency may be either over- or underestimated.

Another important uncertainty is how to define thresholds for vitamin D deficiency or sufficiency in HIV. Current cut-offs (<20 ng/mL or <50 nmol/L for deficiency; 50–75 nmol/L for sufficiency) are based on skeletal health outcomes in the general population. In HIV infection, lower vitamin D levels may also affect immune function and disease progression, raising the possibility that different thresholds should be applied. Furthermore, whether these thresholds can be generalised across different ethnic populations remains unclear [44]. It is also crucial to determine the impact of living in the northern hemisphere on PLWHIV of the Asian and Black populations and whether high doses of vitamin D supplements are needed or not. This may open the door for more research, especially clinical and epidemiological population studies.

## 7. Magnesium and PTH in PLWHIV

The role of magnesium (Mg^2+^) in the regulation of PTH is also important, as vitamin D. As it impacts PTH secretion and function [45,46]. It acts as a cofactor in the synthesis and secretion of PTH. Hypomagnesemia can impair PTH secretion and action, leading to secondary hyperparathyroidism (sHPT) and hypocalcaemia [46,47]. In PLWHIV, factors such as cART (particularly TDF), chronic inflammation, and vitamin D deficiency can exacerbate magnesium depletion, further disrupting PTH regulation [45,48]. Importantly, hypomagnesemia can lead to elevated PTH levels in PLWHIV. For instance, in a study that included 1263 HIV-infected patients with normal kidney function, 16.9% exhibited elevated PTH despite normal or low calcium levels. Low magnesium, low 25-hydroxyvitamin D, and TDF use were significant contributors [45]. Additionally, HIV itself may impair PTH secretion, while TDF can favour hypocalcaemia, potentially resulting in both hypoparathyroidism and secondary hyperparathyroidism [47]. Therefore, monitoring of magnesium status in case of parathyroid dysregulation is recommended. Hypomagnesemia can lead to impaired PTH secretion and action, secondary hyperparathyroidism, BMD loss, increased osteoporosis risk, and potential cardiovascular complications [46,48]. Regular assessment of magnesium, calcium, and PTH levels is recommended in PLWHIV, especially those on TDF, along with appropriate supplementation and dietary interventions to mitigate disturbances and improve bone and mineral health [45,48].

## 8. Overall Impact of HIV on the Parathyroid Gland

### 8.1. HIV Infection’s Direct/Immunological Effects on Parathyroid Function

Parathyroid gland dysfunction is multifactorial and remains under-recognised in clinical practice with regard to HIV infection and cART regimen [49,50,51,52]. In this review, we have highlighted the emerging evidence regarding parathyroid dysfunction in PLWHIV. The most common of such perturbations are improperly raised or suppressed levels of PTH, which are triggered by a combination of immunological, viral, and drug-related factors [52]. Histopathological and autopsy reports have revealed an increased prevalence of parathyroid hyperplasia in PLWHIV, typically as a result of chronic mineral deprivation (in the form of phosphate and calcium) as well as immune deregulation and subsequent inflammation. [53]. Specifically, one study has suggested that the declining concentrations of PTH in the setting of progressive CD4^+^ T-cell decline are congruent with immunosuppression and may impair parathyroid reserve. The above speculation can be supported by the scientific work of Kuhne et al., where the declining amounts of PTH were directly linked to the loss of CD4^+^ T cells, and a possible immunological cause of parathyroid dysfunction was indicated. These studies suggest that dysfunction of the parathyroid gland in PLWHIV is not a cART-related side effect, but rather a product of alterations in the immune system caused by HIV infection, an indication that is consistent with increased surveillance and early diagnosis [52].

### 8.2. cART Effects (TDF and Others on PTH and Bone Metabolism)

Functional studies have also given more hints on the effects of cART, especially the use of the TDF-based regimen, on the functioning of the parathyroid. An increased level of PTH is typical of PLWHIV on TDF, and research suggests that secondary hyperparathyroidism is the result of parathyroid gland overcompensation. These patients have persistently elevated levels of PTH in spite of normal biochemical indices, indicating dysregulation of calcium–PTH dynamics by cART. The pattern highlights the multidimensional relationship that exists between HIV infection, cART, and the parathyroid axis. The available evidence analysed in the current study indicates that HIV infection and cART can affect the normal functioning of parathyroid glands both directly and indirectly. Nevertheless, the fact that few studies have been carried out and that most of the studies have had small sample sizes, limits the extent to which these findings can be generalised. Nonetheless, these limitations still indicate that there is a pressing need to conduct research on the causes of PTH dysregulation in PLWHIV with a particular focus on the inclusion of parathyroid monitoring in metabolic and endocrine assessment [51,52]. Given these insights, the identification of subclinical PTH dysfunction could offer opportunities for earlier therapeutic intervention [53].

The clinical implications of these findings are significant for the long-term care of PLWHIV. Parathyroid gland dysfunction remains under-recognised in clinical practice. However, the evidence suggests that both HIV infection and cART, particularly TDF, contribute to abnormalities in PTH secretion, independent of traditional risk factors such as vitamin D deficiency or hypocalcaemia.

### 8.3. Nutritional and Biochemical Assessment

As summarised in Table 1, PTH dysregulation may be an overlooked cause of bone mineral loss, neuromuscular symptoms, and secondary hyperparathyroidism in PLWHIV. Consequently, clinicians should consider incorporating routine monitoring of serum PTH, phosphate, calcium, and vitamin D levels into metabolic screening protocols, especially for those initiating or maintaining TDF-based regimens [51,53,54]. Early detection of subclinical parathyroid dysfunction offers the potential for timely intervention, which could prevent long-term complications. Moreover, clinicians must be aware of atypical biochemical presentations, such as elevated PTH with normal calcium, which may point to cART-induced parathyroid dysregulation. This approach would help avoid misattribution to non-specific causes and enable better clinical management of PLWHIV [54].

### 8.4. Clinical Implications of Parathyroid Dysfunction in HIV

The metabolic alterations resulting from parathyroid dysfunction have significant clinical consequences in people living with HIV. For instance, an increase risk of fracture and bone disease. Epidemiological studies have demonstrated that PLWHIV have a 2× to 6× fold increased risk of osteopenia, osteoporosis, and fractures compared to HIV-negative individuals [55]. Vertebral and hip fractures are especially prevalent and lead to increased morbidity and mortality. Furthermore, calcium and phosphate imbalances associated with disrupted PTH secretion lead to imbalances in serum calcium and phosphate. Hypocalcemia, often mild, can cause neuromuscular irritability, paraesthesia, and in severe cases, tetany or seizures [56]. Importantly, cardiovascular implications can be attributed to secondary hyperparathyroidism and vascular calcification, and cardiovascular disease risk in the general population. PLWHIV already have increased cardiovascular risk, and parathyroid dysfunction may contribute to this burden [57].

## 9. Strengths, Limitations, and Future Research Directions

The quality of the evaluated research papers is a significant strength of our assessment. This study was conducted as a narrative review instead of a systematic review and provided up-to-date information on the topic. This narrative review aimed to furnish readers with a thorough evaluation of the issue, including a succinct update on the subject matter. This review addresses an important but often overlooked aspect of HIV management, the potential impact of the virus and its treatment on parathyroid gland function. A key strength of this work is its broad and integrative approach, drawing on evidence from clinical case reports, autopsy studies, and prospective trials to build a clearer picture of how HIV and antiretroviral therapy may disrupt the parathyroid axis. This review attempted to explore both structural and biochemical perspectives and highlights emerging patterns and plausible mechanisms of dysfunction, such as altered PTH secretion in the absence of classic deficiencies. In doing so, it brings attention to clinicians to a field that warrants greater clinical and research focus, especially given the long-term metabolic implications for people living with HIV.

However, several limitations must be acknowledged. The available literature is limited by small sample sizes and a predominance of retrospective and cohort studies, many of which lack long-term follow-up. This restricts the ability to draw firm causal conclusions. Additionally, heterogeneity in study design, population demographics, and outcome measures poses challenges for direct comparison across studies. There is also a notable lack of standardised definitions for parathyroid dysfunction in the context of HIV, which may contribute to inconsistent reporting. Finally, the underrepresentation of histological evidence limits our ability to confirm direct viral or immune-mediated damage to parathyroid tissue. Therefore, future research directions may focus on the (i) bone–parathyroid axis and how they modulate the fracture risk in HIV and how vitamin D supplementation may mitigate such risk; (ii) the need to arrange for longitudinal population studies to assess the vitamin D cut off point that protects against complications in different populations; (iii) longitudinal outcomes of long-term parathyroid dysfunction influence morbidity and mortality in PLWHIV, beyond bone health (e.g., neuromuscular symptoms, cardiovascular outcomes)? Are there differences in outcomes between early versus late detection and the management of parathyroid abnormalities in HIV (iv) screening and monitoring strategies, or optimal frequency and cost-effectiveness of routine PTH and calcium, and vitamin D screening in HIV care? Should PTH monitoring be integrated into standard HIV guidelines, and if so, at what treatment stage? (v) In systematic review and meta-analysis, vitamin D supplementation can improve serum 25(OH)D in PLWHIV. However, the authors recommended that the effect of vitamin D on bone mineral density and PTH needs to be further investigated in larger-scale, well-designed randomised, controlled trials [58]. Interestingly, TDF changes the relationship of 25-OHD to PTH, suggesting that a higher-than-usual target for serum 25-OHD concentration might be needed to reduce PTH and optimise bone health [59].

## 10. Conclusions

Parathyroid gland dysfunction is an under-recognised yet clinically significant complication in people living with HIV, arising from the combined effects of HIV infection, antiretroviral therapy, and chronic immune activation. Further research at the cellular and molecular level, alongside large population-based studies, is needed to improve our understanding of vitamin D, calcium, and magnesium metabolism and their impact on PTH secretion and function in this population. In particular, the precise molecular mechanisms through which HIV infection and immune activation alter parathyroid gland physiology remain to be clarified. Equally important is determining how different antiretroviral agents beyond TDF may directly or indirectly affect PTH secretion and calcium–phosphate homeostasis. Another key question is whether routine incorporation of PTH, calcium, phosphate, and vitamin D monitoring into HIV care could facilitate earlier detection of subclinical disturbances, thereby reducing fracture risk and improving long-term outcomes. Future research should prioritise elucidating the mechanistic pathways underlying HIV- and cART-related parathyroid dysfunction and evaluating targeted interventions to optimise bone and endocrine health in this vulnerable group.

## Figures and Tables

**Table 1:** Mechanisms of parathyroid dysfunction in PLWHIV.

Mechanism	Details	Impact on Parathyroid Function	References
Chronic Immune Activation	Persistent HIV infection triggers immune responses that affect parathyroid glands.	Increased parathyroid cell dysfunction and PTH resistance, leading to abnormal PTH secretion.	[13,14]
Vitamin D Deficiency	Low levels of 25-hydroxyvitamin D are common in PLWHIV.	Vitamin D insufficiency exacerbates parathyroid dysfunction and impairs calcium–PTH regulation.	[4,8]
cART Use (TDF-Based)	Tenofovir disoproxil fumarate (TDF) causes phosphate wasting and interferes with vitamin D metabolism.	TDF induces secondary hyperparathyroidism due to impaired calcium and phosphate balance.	[11,12]
Immunosuppression	Declining CD4 count correlates with decreased PTH secretion in HIV-infected individuals.	Decreased PTH levels observed in PLWHIV with low CD4 counts, implying impaired parathyroid reserve.	[12,13]

**Table 2:** Parathyroid dysfunction in HIV-related case reports.

Case Report	Findings	Clinical Implications
[15]	Severe symptomatic hypocalcaemia with primary hypoparathyroidism despite sufficient vitamin D levels.	Suggests that HIV infection directly impacts parathyroid function, independent of vitamin D levels.
[16]	Persistent hypocalcaemia with suppressed PTH levels in an HIV-positive female patient.	Indicates potential HIV-related parathyroid dysfunction, possibly due to immune dysregulation.
[17]	Autopsy findings show a higher prevalence of parathyroid hyperplasia in HIV-positive patients.	Parathyroid hyperplasia may result from chronic hypocalcaemia, immune dysregulation, and vitamin D deficiency in PLWHIV.

**Table 3:** Impact of cART on parathyroid function in PLWHIV.

ART Regimen	Effect on PTH	Associated Factors	Clinical Considerations	References
Tenofovir (TDF)	Elevated PTH levels are seen, especially in the early stages of ART.	Vitamin D deficiency and impaired calcium metabolism contribute to PTH elevation.	Regular monitoring of PTH and calcium is recommended for PLWHIV on TDF.	[19,22,23]
Abacavir/Lamivudine	No significant changes in PTH levels compared to TDF-based regimens.	The study found no significant differences in vitamin D, calcium, or phosphate levels.	Consider alternative ART regimens for patients with elevated PTH levels on TDF.	[19]
Protease Inhibitors (PIs)	May contribute to further PTH elevation through immune dysregulation.	PIs have been linked to altered vitamin D metabolism, exacerbating PTH dysfunction.	Close monitoring of bone health and PTH is advised when using PIs.	[16,19,20]

**Table 4:** Study characteristics of included evidence on parathyroid dysfunction in PLWHIV.

Author, Year	Design	Population (*n*)	Key Parathyroid Outcomes Measured	cART Context	Main Finding
[8]	Cross-sectional	NR	25(OH)D–PTH relationship	Mixed cART	Abnormal calcium–PTH dynamics; PTH stabilised when 25(OH)D > 75 nmol/L
[14]	Case–control (physiological test)	6 PLWHIV; 10 controls	Basal PTH; hypocalcaemia-stimulated PTH	Pre-cART era	Lower basal and maximal PTH response to EDTA-induced hypocalcaemia in PLWHIV (*p* < 0.04)
[13]	Experimental (IHC)	38 PLWHIV; 38 controls	CD4-like expression in parathyroid tissue	Pre-/mixed	Decreased circulating PTH in PLWHIV; CD4-like molecule expressed in parathyroid tissue (mechanistic link)
[17]	Retrospective autopsy study	102 PLWHIV; controls	Parathyroid histopathology	Mixed cART	Higher prevalence of parathyroid hyperplasia in PLWHIV (22.6% vs. 2.6%; *p* < 0.04)
[15]	Case report	1	PTH, ionised calcium, 25(OH)D	Mixed	Severe hypocalcaemia with primary hypoparathyroidism despite sufficient vitamin D
[16]	Case report	1	PTH, total calcium, phosphate	Mixed	Persistent hypocalcaemia with suppressed PTH; features consistent with primary hypoparathyroidism
[19]	Longitudinal cohort (cART initiation)	51 (TDF/Emtricitabine) = 31; Abacavir/Lamivudine = 26)	PTH, 25(OH)D, Ca, PO_4_	TDF vs. non-TDF	Higher PTH at weeks 4, 24, 36 in the TDF group; no differences in Ca/PO_4_/25(OH)D; TDF/FTC independently predicted PTH ≥ 53 ng/L
[22]	Cohort	56	PTH; Ca; 25(OH)D	TDF vs. non-TDF	Increase in PTH on TDF independent of Ca/25(OH)D (mean diff 3.1 pmol/L; *p* = 0.007)
[23]	Cohort	NR	PTH (by Ca quartiles)	TDF vs. non-TDF	Persistent PTH elevation in all corrected Ca quartiles on TDF vs. non-TDF

## Data Availability

This narrative review and all data are included in this review.
